# ‘I’ve learned I need to treat my characters like people’: Varieties of agency and interaction in Writers’ experiences of their Characters’ Voices

**DOI:** 10.1016/j.concog.2020.102901

**Published:** 2020-03

**Authors:** John Foxwell, Ben Alderson-Day, Charles Fernyhough, Angela Woods

**Affiliations:** a Department of English Studies, Durham University, Hallgarth House, 77 Hallgarth Street, Durham DH1 3AY, United Kingdom; b Department of Psychology, Durham University, Science Laboratories, South Road, Durham DH1 3LE, United Kingdom

**Keywords:** Agency, Alterity, Inner speech, Theory of mind, Creativity

## Abstract

Writers often report vivid experiences of hearing characters talking to them, talking back to them, and exhibiting independence and autonomy. However, systematic empirical studies of this phenomenon are almost non-existent, and as a result little is known about its cause, extent, or phenomenology. Here we present the results of a survey of professional writers (*n* = 181) run in collaboration with the Edinburgh International Book Festival. Participants provided detailed descriptions of their experiences of their characters in response to a phenomenological questionnaire, and also reported on imaginary companions, inner speech and hallucination-proneness. Qualitative analysis indicated that the phenomenology of the experience of agentive characters varied in terms of the characters’ separateness from the writer’s self and the kinds of interaction this did or did not allow for. We argue that these variations can be understood in relation to accounts of mindreading and agency tracking which adopt intuitive as opposed to inferential models.

## Highlights

• Varied phenomenology of writers’ experiences of characters’ voices.
• Important point of comparison for experiences of non-actual agents.
• Automatized use of personality models suggesting non-inferential theory of mind.

## 1 Introduction

Engaging with fictional characters is a complex cognitive act which involves the interaction of a range of psychological processes, from mental imagery, to empathy, to theory of mind (Waugh, 2015, Oatley, 2012, Keen, 2006, Zunshine, 2006). Particularly intriguing – and difficult to account for – is the experience which frequently emerges from the creation of fictional characters. A large number of writers report vivid experiences of ‘hearing’ their characters talking to them, talking back to them, and exhibiting an atypical degree of independence and autonomy (Watkins, 1986, Taylor et al., 2003, Porter Abbott, 2011):

Just as summer was ending, one or more of my characters – Celie, Shug, Albert, Sofia, or Harpo – would come for a visit. We would sit wherever I was, and talk. They were very obliging, engaging, and jolly. They were, of course, at the end of their story but were telling it to me from the beginning. Things that made me sad, often made them laugh. Oh, we got through that; don’t pull such a long face, they’d say (Alice Walker, 1983, p. 359)

I always wondered about authors who told me that their characters took on a life of their own. I used to think they sounded a bit pretentious, but then I found out it’s true. I’ll find that a minor character suddenly begins to appear where she wasn’t plotted to be, clamouring for more attention and a meatier part in the narrative. (Rosie Blake, 2019)

It does seem – and I realise this is a psychological trick and it sounds very coy – but it is as if they are speaking and leading those lives. It’s a very symbiotic relationship. You do seem to be with people who have minds of their own, thoughts of their own, but at the same time you’re very much involved in leading their lives with them. (Michael Frayn, 2011)

Understanding what exactly such experiences entail, and why they occur, is therefore of importance to the understanding of how human beings can think about and relate to entities that lie outside of immediate and shared perceptual experience.

As an imaginative activity, the experience of fictional characters has often been understood in relation to the activities of play and make-believe, from the perspective of both readers (Walton, 1990) and writers (Watkins, 1986). Within this framework, characters find their analogue in childhood imaginary companions, which can similarly be experienced by their creators as wilful and independently minded. In their study of 50 writers, Taylor et al. (2003) found that 92% experienced what they termed the Illusion of Independent Agency (IIA), and that their sample scored significantly higher than general population norms on Bernstein and Putnam’s (1986) Dissociative Experiences Scale. Taylor et al. therefore suggest that both imaginary companions and the IIA are the result of children and writers (respectively) practicing imaginative pretence with such frequency that it becomes ‘automatized’, thus leading to a loss of awareness of conscious agency for these activities.

As a result of these findings, Taylor and Mannering (2007) further suggest that writers and children with imaginary companions could be considered ‘expert pretenders’, especially given what they refer to as the frequency with which writers report ‘the experience of characters becoming almost real’ (Taylor & Mannering, 2007, pp. 240, 239). However, in their study Taylor et al. did not appear to separate the experience of characters’ agency and the experience of them as quasi-perceptual, whereas more recent theories of agency-tracking of non-actual agents suggest that there are good reasons for making just such a conceptual separation (Wilkinson & Bell, 2016). Moreover, having had an imaginary companion during childhood did not associate with or predict a high degree of IIA (Taylor et al., 2003), and figures from a more recent study (Fernyhough, Watson, Bernini, Moseley, & Alderson-Day, 2019) suggest that Taylor et al.’s sample of writers did not report a higher rate of imaginary companions during childhood than is typical in an adult population sample. Therefore, although there appear to be some theoretical advantages to drawing parallels between children’s imaginary companions and the fictional characters of adult writers, evidence for a causal or trait link between the two is yet to be established.

Alternatively, writers’ experiences of their characters – particularly of their characters’ *voices* – could be understood according to certain models of inner speech. As verbal thought or ‘the subjective experience of language in the absence of overt and audible articulation’ (Alderson-Day & Fernyhough, 2015, p. 931), inner speech would appear to encompass imaginative experiences of others’ speech, at least according to those models which view it as being essentially ‘dialogic’ and containing ‘other people’ (Vygotsky, 1987, Fernyhough, 1996). Here an analogue can be found in readers’ experiences of fictional characters, which also often involve vivid imaginative experiences of ‘hearing’ characters’ voices (Vilhauer, 2016, Alderson-Day et al., 2017). Alongside these experiences of auditory imagery, many readers infer the mental states of characters and impute intentionality (Dixon and Bortolussi, 1996, Herman, 2008). Although the processes underlying auditory imagery and social cognition are separable, readers’ experiences of fictional characters appear to provide another instance of overlap between the two. In light of the possible developmental relationship between inner speech and theory of mind (Fernyhough & Meins, 2009), it is perhaps not surprising that the voice of the character (auditory imagistic) and the sense of the character as an independent agent (social-cognitive) should overlap in this fashion. However, from writers’ anecdotal accounts of their characters’ voices, it is difficult to tell whether they consider these voices to be a part of, or noticeably distinct from, their own ordinary inner speech.

Given that writers’ descriptions of their characters’ voices often appear to refer to experiences that are not typically shared by the majority of the population, it is also perhaps unsurprising that both cultural stereotypes and creativity research have associated writing with psychopathology (Bentall, 2003; Sass, 2001, Barrantes-Vidal, 2004). In line with this approach, the voice of the fictional character has its analogue in auditory verbal hallucination (AVH), which several prominent theories have associated with misattributed inner speech (Frith, 1992, Feinberg, 1978, Bentall, 1990). However, although the descriptions which writers have given of their experiences of their characters might sometimes appear similar to descriptions of hallucinations, the phenomenology of these forms of experience deserves further investigation to avoid a simplistic or facile identification between them. Furthermore, while it has been suggested that hallucinatory experiences form a continuum with ‘ordinary’ psychological functioning (Slade & Bentall, 1988), the extent to which hallucination-proneness correlates with aspects of imaginative experience (e.g. vividness of mental imagery) has not been established conclusively (Bentall, 1990, Barrett, 1993, Aleman et al., 1999).

Writers’ experiences of their characters provides an important point of comparison and contrast with other forms of experience around which psychological models are constructed. However, the plausibility of any of these explanatory models – and the subsequent implications for theories of cognition – is ultimately dependent on what is *meant* when writers report their characters ‘talking back’, and what such experiences entail. Although there is a substantial amount of anecdotal evidence for this phenomenon, accounts can vary significantly, and it is sometimes difficult to determine the extent to which the writer in question is providing an intentionally literal or figurative description of the experience. The phenomenon has not been extensively investigated empirically, with only one large-scale study (Taylor et al., 2003) and one small-scale study (Doyle, 1998) that we know of.

The present study therefore set out to explore writers’ experiences of their characters’ voices in a detailed and systematic fashion using qualitative and quantitative approaches. To investigate this phenomenon, we collaborated with the Edinburgh International Book Festival to survey the large number of writers who were in attendance. Unlike all previous large-scale studies on writers to date, our sample was limited exclusively to those who were published and who had achieved sufficient professional success to warrant an invitation to one of the world’s largest and most prestigious literary festivals. The majority of our respondents specialised in fiction (77%), and particularly textual fiction (66%), though other forms and genres (e.g. poetry, non-fiction) were represented in the festival and in our sample.

The first aim of the study was to gather qualitative information on writers’ experiences of their characters’ voices, in order to shed light on what exactly writers meant when they reported ‘hearing’ their characters and having characters who ‘talked back’ to them. To this end, the survey contained a detailed phenomenological questionnaire on multiple aspects of the writing experience, including questions on dynamics, inner speech, dialogue, and agency.

The second aim of the study was to determine whether there were any differences between those writers who did and those who did not report ‘hearing’ their characters’ voices. To this end, the study also included a questionnaire on imaginary companions, a measure of everyday inner speech experiences (the Varieties of Inner Speech Questionnaire; McCarthy-Jones & Fernyhough, 2011), and a short measure of auditory hallucination-proneness (the Launay-Slade Hallucination Scale – Revised; Bentall & Slade, 1985). Based on the proposed associations between these concepts and hearing characters’ voices, we anticipated that writers who reported hearing their characters’ voices would display elevated rates of vivid inner speech and hallucination-proneness, and be more likely to have had an imaginary companion during childhood.

## 2 Methods and materials

### 2.1 Participants

Writers attending the 2014 Festival and the 2018 Festival were invited to take part in the survey via an email from the festival organisers, which expressed the aim of developing ‘a better understanding of the processes of literary creativity and in particular the ways writers and storytellers hear and interact with the voices of their characters’. The survey was not open to the general public. Of the 1486 guests invited to the festival across both years (including illustrators, artists, celebrities and public figures promoting books), a total of 181 writers (12%) took part in the survey (61% F; 37% M; 2% Other), with respondents coming primarily from the UK (82%). Participants were asked to choose a description of the form they specialised in (e.g. Fiction (Young Adult/Children’s)) from a list of seven options (see Table 1 for demographic details). The survey was live for five weeks in 2014 and six weeks in 2018. All procedures were approved by the ethics committee of a local university.

**Table 1:** Demographics for the combined 2014 and 2018 samples.

	*Frequency* (n = 181)	*%*
*Age*		
18–24	1	0.6%
25–34	18	10%
35–44	40	22%
45–54	47	26%
55–64	49	27%
65–74	22	12%
75 or over	3	2%

*Country (top 5 listed)*		
UK	147	82%
USA	9	5%
Ireland	4	2%
India	3	2%
Australia	2	1%

*Form*		
Fiction (Adult)	74	41%
Fiction (Young Adult/Children’s)	46	26%
Non-Fiction	29	16%
Other (e.g. graphic novels)	16	9%
Poetry	11	6%
Writing for Performance	4	2%
Storyteller in an Oral Tradition	0	0%

We did not exclude poets and non-fiction writers as several responded to the survey in relation to fiction they had also written, or responded in a way that clearly demonstrated the relevance of the questions to poetry (e.g. writing in the voice of a fictional character) and non-fiction writing (e.g. historical biography).^1^

### 2.2 Measures

The survey was divided into three parts. Section 1 – the Writers’ Inner Voices Questionnaire – specifically asked about participants’ experiences of their characters during writing. Section 2 asked about imaginary companions. Section 3 included the questionnaire items on inner speech and auditory hallucination proneness.

#### 2.2.1 Writers’ inner voices questionnaire

A phenomenological questionnaire was devised for the study, informed by Taylor et al.’s (2003) survey of writers and Woods, Jones, Alderson-Day, Callard, and Fernyhough’s (2015) survey of voice-hearers. All questions apart from 2, 3, and 4 required free-text responses (no word limit); questions 2, 3, and 4 were followed by free-text-response sub-questions if they were answered positively.

1. How do you experience your characters?
2. Do you ever hear your characters’ voices?
2.i. [If yes] Please try to describe what it is like to hear your characters’ voices.
2.ii. [If yes] How, if at all, are these experiences different from your own thoughts or inner speech?
2.iii. [If yes] How, if it all, are these experiences different from hearing the voice of someone who is present in the room?
3. Do you have visual or other sensory experiences of your characters, or sense their presence?
3.i. [If yes] Please tell us about these experiences.
4. Can you enter into a dialogue with your characters?
4.i. [If yes] Please tell us about these dialogues.
5. Do you feel that your characters always do what you tell them to do, or do they act of their own accord?
6. How does the way you experience your characters’ voices feed into your writing practice? Please tell us about this process.
7. Once a piece of writing or performance is finished, what happens to your characters’ voices?
8. If there are any aspects of your experience of your characters’ voices or your characters more broadly that you would like to elaborate on, please do so here.
9. In contexts other than writing, do you ever have the experience of hearing voices when there is no one around? If so, please describe these experiences. How do these experiences differ from the experience of hearing the voice of a character?

#### 2.2.2 Imaginary companions questionnaire

The questionnaire on imaginary companions consisted of three categorical questions. If respondents answered positively, the question was followed by a text-box and an invitation to describe the experience further. The three questions were:

1. Did you have an imaginary friend or friends when you were growing up?
2. Do you have an imaginary friend or friends now?
3. If you ever had an imaginary friend or friends, did they sometimes act of their own accord (as opposed to always doing what you told them to do)?

#### 2.2.3 Varieties of inner speech questionnaire (McCarthy-Jones & Fernyhough, 2011)

The VISQ is an 18-item questionnaire relating to the phenomenological characteristics of inner speech (McCarthy-Jones & Fernyhough, 2011). It includes four factors: *condensed inner speech*; *dialogic inner speech*; *other people in inner speech*; and *evaluative/motivational inner speech*. Participants rated their agreement with the statements provided (e.g. ‘I hear other people’s voices nagging me in my head’) on a 7-point Likert scale.^2^ Each subscale has good internal reliability (all Cronbach’s alpha > 0.70; Alderson-Day et al., 2014, McCarthy-Jones and Fernyhough, 2011).

#### 2.2.4 Launay-Slade hallucination scale – Revised (Bentall & Slade, 1985)

A short 5-item version of the LSHS was used to assess proneness to unusual auditory experiences (Bentall and Slade, 1985, Morrison et al., 2000). Participants rated their agreement with the following five statements relating to atypical auditory phenomena:

1. I hear a voice speaking my thoughts aloud.
2. I hear the telephone ring and find that I am mistaken.
3. I hear people call my name and find that nobody has done so.
4. I can hear music when it is not being played.
5. I have had the experience of hearing a person’s voice and then found that there was no one there.

Although it is a short measure, the 5-item LSHS has been shown to have a moderate/good internal reliability (Cronbach’s alpha = 0.69; McCarthy-Jones & Fernyhough, 2011).

### 2.3 Qualitative coding

The responses from Section 1 were coded using an inductive thematic analysis (Braun & Clarke, 2006). Two raters (JF and AW) developed a set of descriptive codes from the dataset as a whole. Multiple iterations of the coding framework were discussed by the authors before the final version was applied to 20% of the dataset for independent coding by each rater. Once satisfactory inter-rater reliability had been reached (*k* = 0.79), the remainder of the dataset was independently coded by JF, with ambiguous cases flagged for discussion.

The coding scheme reflected four major themes in the data: firstly, concerning the *dynamics* of the experience, relating to how the writer experienced his/her characters; second, how the characters’ voices related to the writer’s own *inner speech*; third, if *dialogue* with characters did occur, how such dialogue was experienced; and finally, if characters did appear to exhibit *agency*, how and when that agency appeared (see Table 2 for a full list of code definitions and frequencies). The *inner speech* codes, *dialogue* codes, and *agency* codes were exclusive – respondents could only receive one code from each of these code groups. All other codes were non-exclusive, although each respondent could only receive each code once.

**Table 2:** Code definitions and frequencies.

*Code*	*Description*	*Example*	*Frequency (n = 181)*	*%*
*Dynamics*				
Hear Characters’ Voices	Responded ‘Yes’ to Q.2	[N/A]	114	63%
Visual/Other Experience of Characters	Responded ‘Yes’ to Q.3	[N/A]	102	56%
Experiential Overlap	Any mention of exploring the storyworld through the character’s senses, and/or of inhabiting/being inhabited by the character	‘I feel like I can get inside their heads and see the world through their eyes, hear it through their ears.’	40	22%
Observation	Any reference to the experience being like watching a film or play, listening to the radio, taking dictation, transcribing, eavesdropping, etc.	‘I have a very vivid, visual picture of them in my head. I see them in my imagination as if they were on film – I do not see through their eyes, but rather look at them and observe everything they do and say.’	57	32%
Felt Presence	Any report of feeling the presence of the character in the real world (i.e. in relation to the writer’s actual body)	‘Sometimes, I just get the feeling that they are standing right behind me when I write. Of course, I turn and no one is there.’	36	20%
Physical Acting Out	Any mention of actually voicing/doing what the character says/does	‘I try and embody them a little bit in the privacy of my own home or when I’m alone – this is usually by speaking as them out loud, and occasionally acting stuff out a bit too physically.’	20	11%
Experience of Characters Post-Novel	The writer continues to hear the voices of/experience the agency of characters after the text (e.g. novel) is finished	‘Sometimes they hang around for a while. I can’t rush from one book to another. I need time for them to leave.’	66	37%
Actual Hallucinations	Any report of voices or visions as literally hallucinatory, whether related to writing or characters or not (e.g. accounts of psychotic episodes, sleep-related hallucinations, etc.)	‘I did so [hear voices when there was no one around] when I was younger (under 16, and only when alone) which troubled me very slightly. I have long since realised that this is quite usual.’	38	21%

*Inner speech*				
Fully Distinct from Inner Speech	Any report of characters’ voices being clearly distinct from the writer’s inner speech, not merely because of its auditory properties but because it ‘belongs’ to the character/is not felt to be owned by the writer	‘They do not belong to me. They belong to the characters. They are totally different, in the same way that talking to someone is different from being on one’s own.’	59	33%
Not Fully Distinct from Inner Speech	No clear sense of characters’ voices being distinct from inner speech – usually contradictory, or explicitly referring to lack of distinction (or impossibility of making such a distinction)	‘I’m not sure there’s any qualitative difference for me between [characters’ voices and] my own thoughts/inner speech because if you write fiction you spend a lot of time putting yourself into somebody else’s shoes, and somebody else’s voice.’	55	30%

*Dialogue*				
Dialogue as Self	The writer can and does enter into direct dialogue with the character without abandoning his/her role as author (i.e. not being or speaking as another character)	‘I tend to celebrate the conversations as and when they happen. To my delight, my characters don’t agree with me, sometimes demand that I change things in the story arc of whatever I’m writing’	27	15%
Dialogue as Character	The writer only enters into dialogue with character(s) by taking on the role of the character and/or another character as interlocutor	‘[I]t’s never me talking to the character, it’s me being another character talking to that character. As if it’s an improvised play and I’m playing all the parts.’	15	8%
Dialogue as Possible	The writer acknowledges the possibility of being able to enter into a dialogue with characters, but doesn’t practice this	‘I know I could because I know the characters but it hasn’t happened. If I wanted to ask any of them what they were thinking I could and they would tell me.’	14	8%

*Agency*				
Full Agency	At least one character is experienced as fully agentive without any indication of variability	‘They do their own thing! I am often astonished by what takes place and it can often be as if I am watching scenes take place and hear their speech despite the fact I am creating it.’	23	13%
Temporally Emergent Agency	Any reference to needing to be at a certain point in the story before the character exhibits agency	‘They generally do as they are told! When sets of characters have matured, however, they sometimes seems to take on an independent existence and can surprise me with how they interact.’	48	27%
Unspecified/Occasional Agency	Characters’ agency is reported, but without being either continuous or temporally emergent	‘The ‘yes’ here is equivocal but more accurate than ‘no’… it is not something that happens all the time. When it does it is vivid.’	40	22%

## 3 Results

### 3.1 Dynamics

Almost two thirds of writers in our sample reported hearing their characters’ voices (63%). In the majority of cases, this was clearly related to the sense of the character’s voice or appearance having distinctive characteristics (e.g. accent, gender, etc.).

*I hear them in my mind. They have distinct voice patterns and tones, and I can make them carry on conversations with each other in which I can always tell who is ‘talking’. (R 35)*

Often respondents would make explicit reference to their characters’ voices being imaginary, ‘in the head’, or ‘in the mind’s eye’; very few respondents suggested that their characters’ voices were the same as the voices of people actually present in the room. Over half of our sample (56%) reported visual or other sensory experiences of their characters, although responses varied greatly in terms of the ‘completeness’ of such imaginings:

*I do sometimes see them, their bodies in particular; a way of standing, or turning, or another action. But I rarely, if ever, see the faces of my characters fully formed, hardly ever. (R 145)*

Moreover, although 11% of respondents reported having felt the presence of their characters, this was usually described as occurring in the absence of any visual sense of the character:

*I sense their presence as you sense somebody in a dream. They are very much known to me but only in peripheral vision and as an atmosphere or a force exerting itself. I wouldn’t be able to sit opposite a character, so to speak, and see them, talk to them etc. They aren’t something that can be interrogated or pinned down. (R 51)*

Those writers who did not endorse hearing or seeing their characters sometimes gave explicit reasons for providing negative responses. Usually, the reason given was one of the following three: the writer was too conscious of embodying the characters’ voices to attribute those voices to the character; the writer experienced their characters more as narrative props or functions than personified agents; or the writer interpreted the question literally, and reserved ‘hearing’ and ‘seeing’ for experiences in other modalities:

*Having found them, it’s then my job to embody them, which includes embodying their speech. This is not like listening, for me, nearly as much as it is like reading aloud […] I’m performing the people, I’m dramatising them (R 57)*

*I tend to think of my characters as narrative constructions rather than real people […] I don’t ‘hear’ those voices in my head. (R 59)*

*Variable – may be via any sense, but the sense is not experienced as real or in real time – more at the level of intensity that would be there in a memory (R 73)*

Although respondents were not asked directly about experiential overlap, physical acting out, and observation, all three featured in writers’ attempts to describe how they experienced their characters. 22% of our sample reported that their imaginative experience of the storyworld occurred through their characters’ senses, as if they were sharing a physical body. In a few cases this was explicitly associated with being unable to see a character’s face:

*If the character feels something I feel it, whether emotional or sensory. (R 40)*

*I often don’t see their faces precisely. Sometimes because I’m the character and I’m looking out, but often because I don’t really need to unless it’s important and I have to decide what they look like. It’s more like a dream in that sense. (R 38)*

Physical acting out, which involved actually performing or rehearsing the speech and actions of characters, was reported by 11% of our sample. Often this feature was described as serving a distinct purpose:

*I’ll play-act a dialogue between characters, in order to map out a scene in my head. (R 56)*

Observation, on the other hand, tended to involve a sense of separation between writer and character, at least insofar as it involved watching or listening to characters from an external perspective. Often the writer would describe themselves as ‘just’ transcribing or recording events which they imagined observing, although in some cases the film or play metaphor used was extended to include their role as a ‘director’ or ‘editor’:

*I can watch them going about their business in a kind of inner cinema screen often complete with dramatic score […] I find the imagined dialogue relatively easy to write it seems as if I just have to transcribe what they say. I can also rewind the inner tape and listen again if necessary (R 74)*

*I usually experience them as if I’m watching a disjointed film that I can play forwards and backwards, making small (or huge) changes to each scene and seeing alternative endings unfold in real time. […] I edit as I write, so the experience is of playing conversations as if using a suite of video-editing software – spooling forward and backwards. (R 168)*

However, despite the apparent incompatibility of experiential overlap and observation, some respondents reported both kinds of experience. Usually this was because the writer referred to different experiences at different times, or experiences which related to different characters:

*There is usually one character (always a central one) who feels like ‘me’ and I experience them from the inside out. Other characters I observe rather than inhabit. (R 114)*

Over a third of writers reported experiencing their characters’ voices after having finished working on the narrative in which they appeared. Often these experiences were described as becoming increasingly attenuated or infrequent as time went by, and/or in terms of the characters being ‘replaced’ by characters from a new work. In a few cases, however, the characters persisted to such an extent that they affected or interfered with the writer’s new projects:

*They live on but not in such a pressing way; they get superseded by the voices of other characters. (R 114)*

*They vanish mostly. Occasionally, with strong characters they come back later and mess up something that’s coming afterwards. A few of them will never go but they don’t get in the way. (R 120)*

Finally, hallucinatory and hallucination-like experiences reported by writers in our sample (21%) varied considerably in their form and content. Most common were hypnagogic and hypnopompic hallucinations (experienced while falling asleep or waking up), followed by single hallucinatory experiences and/or hallucinatory experiences during childhood. Some respondents who reported hallucinatory experiences stated that there was a noticeable qualitative difference between these experiences and their experiences of their characters.

*I have experienced hypnagogic hallucinations […] These have mostly been aural in character and it’s a lot like eavesdropping in on conversations (the voices are never talking directly to me) […] I do actually hear them vividly, which is why I can be so emphatic about not hearing my characters at all. I have never heard them in this same physical way. (R 106)*

*I’ve had strong and convincing experiences of the presence of God. At least, I think I have: I described them, with the fatal consequence that it’s now difficult to remember what it was like apart from my description. Apart from the sense of needing to attend as purely and patiently as possible, without pre-emptive ordering that might close down what’s happening, I don’t think these experiences have very much in common with my experiences with characters. A different degree of otherness was involved. And I never heard a voice. (R 57)*

Very few writers reported hallucinatory experiences which were in any way related to their characters:

*I have heard character’s voices when I’ve been under extreme duress as well – once on a mountain, feeling as if I was unable to go forward or back, almost frozen with terror and vertigo, I clearly heard the voice of one of my characters telling me what to do, reassuring me and encouraging me to go on. (R 173)*

### 3.2 Inner speech

Amongst those writers who did report hearing their characters’ voices, an important distinction emerged concerning the relationship that these writers described between their own inner speech and the characters’ voices. When stating that they heard their characters’ voices, some writers (30%) referred to their awareness of how the character’s voice sounded, or their sense of what the character would say in a given situation. For these writers, the character’s voice was not separate (or not separable) from their own inner speech, either because the character’s voice *was* a part of the writer’s inner speech, or because the character’s voice blurred the boundary between self and other:

*I have a dual experience – I still have my own POV [point of view] but I have my characters’ too. (R 144)*

*It’s like when you see a dress in a shop window and you hear your mum’s voice saying ‘it won’t wash*…*’ in your mind. It’s involuntary but not intrusive, and it’s not like hearing ‘real’ voices. Something like an invoked memory. […] I suppose it’s a kind of ventriloquism. Ultimately it’s me speaking to myself, but imagining/putting on a different voice to do it. (R 172)*

By contrast, other writers (33%) stated that their characters’ voices *were* clearly distinct from their own inner speech. Often this distinction would be defined in terms of ownership, as if the character were a separate entity.

*They feel ‘embodied’ in a way my own thoughts and interior monologues do not. They have an urgency and an ‘otherness’ – which I can sense rather than explain. (R 22)*

*They have a different voice to my own inner thoughts/speech; I can tell it’s a character and not me, and not just because of the subject matter. Also when my characters are running dialogue in my head I feel like a spectator, but with my own inner speech I feel like the one speaking. (R 122)*

### 3.3 Dialogue

The most common form of dialogue with characters reported by our sample involved the writer speaking with a character directly (15%), with the character as a separate imaginary interlocutor. In some cases this form of dialogue was described as infrequent, or other forms were reported as being more common:

*They sometimes tell me that what I have in mind for them isn’t right – that they would never behave or speak that way. I don’t usually answer back. (R 150)*

*I can ask them questions and they’d answer as if without my input, I haven’t done it much but when I do it works just as a normal conversation would do often times they do go off on a tangent. (R 29)*

The remaining writers who reported engaging in dialogue with their characters were split between dialogue as character (8%) and dialogue as possible (8%). The former group contained responses which referred to the writer needing to take on the role of a character in order for dialogue to occur:

*It’s not really me entering the dialogue, it’s me as another character so I can hear what they would say to each other. (R 104)*

Dialogue as possible, on the other hand, was applied to responses in which the writer stated that they believed they could enter into dialogue with characters but that this was not ever actually practiced:

*I can but I never do it. Still, when they surprise me by bending the conversation they are having with themselves or another character in an unexpected direction I might mutter: ‘So, that is what you are like.’ (R 26)*

### 3.4 Agency

Although the majority of writers in our sample reported characters who exhibited their own agency (61%), there were substantial differences in terms of how and when this experience manifested. It was most common for writers to report that the agency of their characters only emerged after a certain point had been reached during the writing process (26%).

*To begin with they feel under my control and then at that certain point when they feel completely real, it becomes a matter of me following them, hoping to steer. (R 100)*

*I nowadays just plan my books halfway as I know that in the middle of the writing process the characters will take over the story so my planning will become useless anyway. (R 41)*

Although not as common, writers who described experiences of characters’ agency as infrequent or occasional also made up a substantial percentage of our sample (22%). In contrast to cases of temporally emergent agency, these responses gave no indication that the development of the narrative was *necessary* for characters to manifest agency, and therefore tended to implicitly or explicitly describe the phenomenon as unpredictable.

*My characters can often swing the story in an unpredictable direction. Depending on the story, I can either rein them in or let them run with it. (R 152)*

*I LOVE it when my characters go off script. It’s one of my favourite parts of being a writer, and often these unexpected plot twists are the best of all. (R 37)*

Finally, writers who reported characters’ agency without any indication of variability were least frequent in our sample (13%). However, these responses did not necessarily suggest that other aspects of the writing process were not under the writer’s control (e.g. situation, setting, etc.), only that the character was fully in control of their own speech/actions.

*It’s the characters who make the thing happen. I can’t make them do what they don’t want to. (R 17)*

*I don’t think it’s ever a question of my telling them what to do. They just do what they do and I transcribe/describe their dialogue/actions. However, I set the parameters for their existence or activities – decide where they are located, who they are talking to etc. (R 106)*

It is worth noting that several respondents who did not receive an agency code still reported being aware of not always consciously deciding aspects of the narrative and characters’ behaviour (including dialogue), but did not attribute agency to their characters:

*[T]he characters are frames, sets of priorities and emotions with, if you like, narrative vectors. Those vectors in collision with external events and other characters will (must be engineered to) produce the story I want to tell. Sometimes the emergent pattern will take precedence over the plan; other times I will rework characters to push the narrative in the right direction. (R 36)*

*I find that whole thing of ‘my character just took over’ a bit cringey, to be honest. But then I am more of a plotter, so I like to know where I am going with a character. However, you can intend one thing, then find when you actually create the scene or circumstance, that your greater knowledge of your character suggests something better, more in keeping. (R 93)*

Of course, writers who reported that their characters did exhibit agency often implicitly or explicitly affirmed that they knew their characters were imaginary (and aside from non-fiction writers, it was incredibly rare to find responses which positively affirmed any belief in the extra-textual ‘reality’ of characters). However, this awareness did not necessarily prevent the ‘illusion’ of experiencing characters’ agency from occurring.

### 3.5 Case studies – Variations in characters’ agency and alterity

R 157 – Characters exhibit agency and alterity

*[I]n order to feel I really understand a character I have to be able to hear his/her voice in my head as if someone is speaking to me from outside my brain: if I feel like I’m creating what the character says, then the writing is rarely as good and it feels much more like an uphill battle. At the start of a book, I write more slowly and it’s much more painful as I’m still trying to ‘tune’ the characters in. As they start speaking in my head, it becomes easier and the writing speeds up and becomes more fun. […] I often see a ‘movie’ of the book as I write… but one I can move about in like the director, suggesting different dialogue (often a ‘conversation’ with the characters rather than an order) and moving things about as need be until the scene ‘runs’ right.*

*I tend to have a sense of how tall they are relative to me… And though I don’t see faces I do know roughly what they look like. I recognise real people mostly by their hair and specific memorable features so that carries over into what I see of my characters. Sometimes I see what they’re wearing – it depends if it’s important to me and them. I also have a sense of what it would feel like to be in a room with the characters – what their emotional ‘energy’ (for want of a better expression) is like at a given time. But mostly, unlike real people, they never feel like they’re standing too close to me!*

*My characters need to feel separate for me to hear their voices, which also means that when I’m trying to ‘put words in their mouth’ instead of listening they often talk back. And then we discuss things until I find what they would say. If I’m really stuck on the emotional transitions in the story, then listening to what the characters want to say is extremely important. […] I write in a way that’s equivalent to method acting: I have to be the character before I know what to write… and before I can listen to them as separate people in my head.*

They definitely act of their own accord! And it’s usually best to let them. Plotting for me is as much about finding out *how* my Book-People get from A to B as deciding what I want the story to be about at the level of X happens and then Y. This is where a lot of the surprises in my work come from.

This response describes quite a well-developed sense of the character’s agency and alterity (the latter being a term from phenomenology which refers to that which is experienced as ‘not me’ or ‘other-than-me’ (Overgaard & Henriksen, 2019)). Alongside the separateness of the characters’ voices from the writer’s own inner speech, the writer observes her characters, has a sense of the characters’ presence, and sometimes enters into dialogue with characters as if they were separate entities. The characters’ agency is, moreover, explicitly linked to their alterity. What is particularly interesting in this case (and somewhat less common) is the way in which the agency of the characters only emerges after a process of inhabiting their perspectives: the writer shifts over time from ‘being’ certain characters to experiencing the fully-fledged independence of those characters in terms of both agency and alterity.

R 65 – Characters exhibit agency without alterity or additional dynamics

*Once they are present, they engage in the action seemingly without any guidance. I don’t ‘hear’ them but I know what they’re saying, seeing, feeling, and I just write it down.*

*It [characters acting of their own accord] doesn’t happen all the time. It’s mostly to do with dialogue. Snatches of what they say jump into my head. This can progress the story in unexpected ways.*

*Sometimes I struggle to catch their voices and I know I haven’t ‘got’ the character yet. Sometimes I have to haul them back from places where they want to go but I don’t want them to. Sometimes they want to take over the story at the expense of the ‘main’ character and the plot I’m trying to pursue. It’s irritating when the demands of the plot force me to soften, alter, change, expunge a character. It’s very hard to get back into the swing of writing.*

This writer provided negative responses to questions 2 and 3 (‘Do you ever hear your characters’ voices?’ and ‘Do you have visual or other sensory experiences of your characters, or sense their presence?’). In contrast to R 157, the experience of characters does not appear to involve the same kind or degree of quasi-sensory dynamics – instead, characters’ speech and actions are ‘known’ rather than observed. However, characters still appear to manifest agency, sometimes producing dialogue that does not feel consciously created. As with several other cases of occasional manifestations of characters’ agency, the writer acknowledges that the experience has a phenomenological profile which noticeably differs from experiences of characters when they are not manifesting agency. Particularly noteworthy in this response is the implication that these occasional manifestations of characters’ agency do not always take precedence over the writer’s plans for the narrative, since it was more common to find responses which suggested that characters’ actions that were apparently self-willed ought to take precedence over what the writer had consciously decided (i.e. ‘the character is always right’).

R 100 – Characters exhibit agency with additional dynamics but without alterity

*[I experience my characters as] memories and daydreams, like I do people I have met in real life. Sometimes just visual, sometimes just audio, sometimes both.*

*It’s like questions or phrases that pop into my head that show me who the person is. They feel like memories of conversations, very similar to recollections of my real life. […] I am aware that I am generating the voices, even though I’m not sure what they will say. It’s like the best game ever.*

*I often make tea and run conversations between myself and a particular character, speaking both sides. Sometimes I enact my character and roleplay them making the tea and speaking their thoughts.*

*To begin with they feel under my control and then at that certain point when they feel completely real, it’s becomes a matter of me following them, hoping to steer.*

*I speak and write reams and reams of dialogue for everyone in all my stories. Conversations that couldn’t even logistically happen due to time barriers etc. too. I sometimes write a scene and then read it back and it’s almost like the character involved is saying to me, ‘I just wouldn’t do that, are you stupid?’*

*I think sometimes the character’s ‘voice’ can be as much a feeling as actually hearing words. I often picture characters sat waiting, looking at me like they’re saying, ‘Really? Is this what you’re going to make happen?’*

*I often hear the voice of my Grandmother in situations where a nugget of advice she would give me is particularly relevant.*

As this response demonstrates, it was possible for writers to report experiencing their characters in a number of different ways, depending on the technique being used. However, here the writer does not describe his characters’ voices as being necessarily distinct from his own inner speech, in part because his inner speech already contains the voices of ‘other people’. In effect, the boundary between self and other is too blurred to suggest that the writer has any distinct impression of characters’ alterity – at least, not in the sense described by R 157. The forms of ‘interaction’ (i.e. dialogue) engaged in by the writer are also noticeably different, being more akin to a form of self-talk involving different perspectives than a conversation with an imaginary interlocutor. On the other hand, this writer also describes sometimes experiencing his characters in a manner similar to R 65: being aware of a character’s ‘voice’, and the intent behind it, without this necessarily involving an imaginative experience of the relevant (quasi-)sensory properties.

### 3.6 Associations within the writing experience: characters’ voices, inner speech, and agency

To examine these characteristics further, a series of chi-square analyses were run to compare the wider phenomenological characteristics of those (i) who did and did not endorse hearing their characters’ voices, (ii) those who described varying levels of the experience being distinct from their inner speech, and (iii) those who experienced their characters as having a notable degree of agency.

For hearing characters’ voices, associations were tested for the presence of visual characteristics, the feeling that the writer was observing their characters, engaging in a dialogue with characters, and the level of character agency (applying a Bonferroni correction across the four tests to reduce alpha to *p* < 0.0125). Writers who experienced their characters’ voices were significantly likely to also experience their characters visually (χ^2^ = 15.66, *df* = 1, *p* < 0.001), and to feel like they were observing their characters (χ^2^ = 21.84, *df* = 1, *p* < 0.001). Experiencing character’s voices was also associated with them having any agency at all – whether occasionally, emerging over time, or fully (χ^2^ = 21.01, *df* = 3, *p* < 0.001) – but not significantly with levels of dialogue (which failed to survive correction for multiple comparisons; χ^2^ = 9.77, *df* = 3, *p* = 0.021).

Following this, we explored how the codes attributed for inner speech and characters’ voices (i.e. characters’ voices as fully distinct from inner speech, not distinct, or not there at all) picked out different patterns of experience. As above, chi-square tests were applied to our coding for observational experiences, dialogue, and agency, along with the addition of a further code: the sense of experiential overlap between writer and character. Those writers whose characters were fully distinct from their inner speech were significantly likely to report dialoguing as themselves with the character (χ^2^ = 22.19, *df* = 6, *p* < 0.001), to feel like they were observing their characters (χ^2^ = 32.15, *df* = 2, *p* < 0.001), and to experience their characters as possessing full agency (χ^2^ = 28.29, *df* = 6, *p* < 0.001). In contrast, those who reported experiencing their characters’ voices, but without these voices being distinct from their inner speech, were also likely to describe their own experiences ‘overlapping’ with characters’ experiences in some way (χ^2^ = 15.16, *df* = 2, *p* = 0.001).

We applied the same analysis to the coding for agency and its associations with observation and dialogue. Characters experienced as either fully agentic or whose agency emerged over time were significantly likely to be experienced in an observational way (χ^2^ = 13.56, *df* = 3, *p* = 0.004). However, no association was seen between the capacity for dialogue and the characters’ perceived levels of agency (χ^2^ = 13.52, *df* = 9, *p* = 0.140).

### 3.7 Associations with hallucination-proneness, imaginary companions, and everyday inner speech

Finally, we examined whether these distinctions regarding characters’ voices related to experiences outside of writing: namely hallucination-proneness (and specific reports of AVH), the presence or history of imaginary companions, and everyday experiences of inner speech (applying a Bonferroni correction across the seven tests to reduce alpha to *p* < 0.007). Of the sample, 171 respondents completed all of the additional measures.

When writers with and without characters’ voices were compared, the former scored significantly higher for self-reported levels of hallucination-proneness on the LSHS (*t* = 2.826, *df* = 169, *p* = 0.005), although they were not more likely to report specifically AVH-like experiences in the survey itself (χ^2^ = 2.58, *df* = 1, *p* = 0.109). For imaginary companions, only those with (*n* = 65) and without (*n* = 116) ICs during childhood were compared, as too few had current ICs (n = 10); nevertheless, no association was evident between having had an IC in the past or experiencing characters’ voices (χ^2^ = 0.41, *df* = 1, *p* = 0.524). The four subscales of the VISQ were also examined: of these, only the experience of other people in inner speech was significantly elevated in those who experienced the voices of their characters (*t* = 2.863, *df* = 169, *p* = 0.005; all other *p* > 0.250).

In addition to the distinction between writers who did and did not hear their characters’ voices, our qualitative analysis suggested that certain properties hung together, such as characters’ voices being distinct from inner speech, characters being observable, and characters exhibiting agency. In total 25 respondents (15%) had all three properties, constituting a group we defined as ‘high alterity’. Compared with the rest of the sample (and applying the same Bonferroni correction: *p* < 0.007), the high alterity group did score significantly higher on the LSHS (*t* = 3.03, df = 169, *p* = 0.003). There was also a non-significant trend for this group to be more likely to report AVH-like experiences in the survey itself, although this did not survive correction for multiple comparisons (χ^2^ = 5.35, *df* = 1, *p* = 0.021). However, they were not more likely to have had an IC in the past, and the quality of their inner speech did not differ significantly from the rest of the sample.

## 4 Discussion

The first aim of this study was to survey the phenomenological qualities of writers’ experiences of their characters, particularly in relation to writers’ reports of ‘hearing’ characters’ voices. Although ‘hearing’ the voices of characters and experiencing characters as agentive were both fairly common amongst writers within our sample, these experiences were also varied and complex. The descriptions provided by respondents highlighted the multiple ways in which a character might be ‘heard’, ‘talked to’, and experienced as exhibiting independence.

Above all, the responses suggested an important distinction relating to whether (or not) characters manifested *alterity*, i.e. the extent to which they were experienced as separate or apart from the self (Overgaard and Henriksen, 2019, Zahavi, 1999). While it could be argued that, broadly speaking, *any* imagining of an ‘other’ necessarily entails a minimal sense of alterity, what we are concerned with here is the more pronounced sense of separation suggested by some of the responses. The features of writers’ experiences of their characters which indicated this latter sense of alterity include the experience of characters’ voices as distinct from the writers’ own inner speech (coming from outside the bounds of the self), and the experience of characters as capable of being observed or interacted with (separation from the self typically being a precondition for such activities). By contrast, other writers clearly did not experience their characters as exhibiting this form of alterity, often because their responses suggested a blurring of the boundary between self and other, or else because the character did not manifest the necessary features which would make interaction possible.

Those responses which indicated the alterity of characters would appear to support the analogy drawn between characters and imaginary companions (Watkins, 1986, Taylor et al., 2003), since these writers appeared to experience their characters as entities to be interacted with, or at least as manifesting a kind of alterity that would allow for such an interaction. In other words, for these writers, characters are experienced as sufficiently separate from the self to justify the comparison of a character to an imaginary ‘companion’, as opposed to being simply an imagining without this kind of external-to-self dimension. Of course, as with the imaginary companions of children (Taylor & Mottweiler, 2008), this does not necessarily entail any belief in the reality of the characters on the part of the writer.

However, while there was a strong association between codes which suggested the alterity of characters and the sense of characters’ agency, the number of writers who reported characters’ agency was almost double that of writers whose responses indicated a sense of the characters’ alterity. Therefore, a large number of writers who did not observe their characters and who did not hear their characters’ voices as distinct from their own inner speech (or who did not hear their characters’ voices at all) still described their characters as exhibiting agency. This variation suggests that we should be wary of assuming an automatic (albeit intuitive) conflation of the two senses of ‘independence’ (as ‘separate’ and as ‘free’), especially since descriptions of characters’ agency did not necessarily refer to an imaginative experience of hearing or seeing the character as another agent *in action*. Instead, the experience of characters’ agency was often described as a sense of tension between the character and the plot, as a sudden awareness of ideas for the plot or snatches of dialogue which did not feel consciously determined, or as a sense of knowing *how* the character would behave in a given situation without having to consciously *decide* how the character would behave. Such experiences still appear to involve the sense of another’s agency, even if not accompanied by any concomitant sensory imaginings of that other in action. Note that this does not mean that sensory properties of characters are never imagined by these writers, but only that such quasi-sensory experiences do not appear to be a necessary feature of the experience of characters’ agency.

A possible parallel with such experiences of characters’ agency is to be found in voice-hearers’ descriptions of ‘soundless voices’, which involve a sense of receiving a message or meaning without any accompanying auditory properties Bleuler, 1911, Janet, 1889, Jones, 2010, Larøi et al., 2012. While these experiences might differ substantially in other respects, one possible area of overlap is the apparent experience of another’s agency without any accompanying sensory or quasi-sensory experience of that other. Accounting for this phenomenon, Wilkinson and Bell (2016) argue for the non-reflective (or ‘non-inferential’) detection and tracking of the agency of others (Wilkinson & Bell, 2016). According to Wilkinson & Bell’s model, the form and modality of the experience of the other is incidental to the detection and representation of the other’s agency, given that human beings appear to be evolutionarily biased towards detecting and representing agents in their environments (Barrett, 2000), and that contingency of behaviour appears to play a far more important role in agency attribution than the perceptual features of a perceived object (Johnson, 2003). In effect, since the representation of an agent is not necessarily inferred from perceptual or quasi-perceptual phenomena which we know to be usual properties of agents (e.g., a voice, a body, a human face, etc.), the sense of the other’s agency is not necessarily dependent on an experience of the agent *per se*.^3^ Indeed, the potential incorrigibility of our experiences of agents – the fact that we can still *experience* something as an agent even when we do not *believe* that it is an agent (Johnson, 2003) – not only suggests that agent detection and representation is intuitive rather than inferential (Wilkinson & Bell, 2016), but also appears particularly relevant to the ‘illusion’ of characters’ agency. According to this model, it is conceivable that the writer’s lack of conscious awareness of their own agency – such as might result from the automatic (Taylor et al., 2003) or emergent (Bernini, 2014) choices made during the writing process – could generate the illusion of characters’ agency even *without* being accompanied by any additional phenomenological features pertaining to those characters.

Another potentially similar experience is reported in Alderson-Day et al.'s (2017) study of readers, in which nearly a fifth of respondents described unbidden experiences of characters outside the context of reading. While this ‘experiential crossing’ sometimes involved simulations of a character’s speech, it more frequently contained instances of ‘mindstyle’ (Fowler, 1977, Semino, 2007) – a term from cognitive stylistics which refers to the linguistic rendering of the worldviews and cognitive habits of a particular character – or the more general deployment of a character’s ‘consciousness frame’ (Palmer, 2004) – essentially a schema for the character’s perspective constructed during reading. As such, experiential crossing more often included characters’ thoughts and feelings as opposed to specifically quasi-perceptual elements, being ‘more like a habit of thinking or expectation of *what* a character would say in a given situation’ (Alderson-Day et al., 2017, p.107). Like writers’ reports of characters’ agency, readers’ awareness of the characters’ thoughts, feelings, or speech in response to extratextual situations appeared to be non-reflective, as opposed to being reached through deliberative empathising or reasoning about the characters’ mental states. As Alderson-Day et al. suggest, such experiences could be examples of the generation and deployment of ‘personality models’ used to predict (real-world) others’ behaviour in potential scenarios (Hassabis et al., 2014). This understanding of experiences of characters’ agency could be expanded to include those writers who *do* hear their characters’ voices when considered in relation to certain theories of inner speech. According to a Vygotskian approach, the internalisation and articulation of others’ perspectives in dialogic inner speech make up an important part of higher cognition (Fernyhough, 2008). Moreover, this does not only manifest as a more abstracted kind of perspective-taking; as the VISQ attempts to measure, there are varying degrees to which individuals are aware of their inner speech incorporating other people’s voices (McCarthy-Jones & Fernyhough, 2011).

According to this approach, the experience of characters ‘acting of their own accord’ is not necessarily different in kind from our imaginings and predictions of the behaviour of real people, including the conscious awareness of other people’s voices in inner speech reported by some individuals. The sense of characters’ agency may arise because this experience is more noticeable and thus more noteworthy than automatically generated imaginings of real people, because (a) characters are fictional entities, and so the sense of their agency is arguably not diminished through comparison with the more conspicuous agency of a real individual with whom they share an identity, and (b) the later experience of automatic response generation which develops after a certain point *contrasts* with the experience of having to consciously decide how the character responds in the earlier stages of character creation. The preponderance of responses which described characters’ agency as temporally emergent or only occasionally manifesting would appear to support this notion, since these writers appeared to be aware of losing the sense of reflectively deciding how their characters acted. In effect, what becomes automatized is therefore not so much the general activity of pretence *itself* (Taylor et al., 2003), but is instead the automatic prediction of particular characters’ responses to situations on the basis of having become familiar with their mental functioning, just as happens when we become better acquainted with real people (Wittgenstein, 1980, Hobson, 2002, Herschbach, 2008, Thomas and Fletcher, 2003). Indeed, several writers who described their characters’ agency as temporally emergent made this comparison explicitly (e.g., ‘There comes a point when that character is imaginatively real and I know them like you’d know a real person you’d met and consequently I can anticipate their behaviour’). Another parallel could be identified here with readers’ reports of experiential crossing, which similarly appear to involve the automatic deployment of a character’s consciousness frame (Alderson-Day et al., 2017). The extent to which such experiences of characters’ agency also contain simulations of characters’ voices might conceivably vary in the same way that people can be aware of others’ perspectives or personality models being more or less fleshed out as ‘voices’ within their own everyday inner speech.

However, there may still be an important distinction to be drawn between writers who did and did not experience their characters with a high degree of alterity. As a whole, our sample of writers did not display any significant differences from other population samples on the three additional measures we included: prevalence of imaginary companions during childhood (Fernyhough et al., 2019); the quality of inner speech on the VISQ (McCarthy-Jones & Fernyhough, 2011), with the exception of the subscale which measures the experience of other people in inner speech (in which writers who heard their characters’ voices received higher scores); and hallucination-proneness on the LSHS (McCarthy-Jones and Fernyhough, 2011, Alderson-Day et al., 2017). Similarly, comparisons between writers who did and did not hear their characters’ voices showed no differences on the first two measures, apart from the ‘other people in inner speech’ subscale. Since respondents might have included their experiences of their characters’ voices as ‘other people’ in their inner speech, we are wary of assigning any importance to this difference, especially given that inner speech did not appear to differ in relation to any of the other subscales. However, those writers who heard their characters’ voices did receive a higher score on the LSHS than those who did not, while the group we defined as ‘high alterity’ (receiving the fully distinct from inner speech, observation, and agency codes) scored higher still. It is possible that more pronounced experiences of characters’ alterity are therefore related to a greater proneness to experiencing one’s imaginings in general as perceptual or quasi-perceptual phenomena. One important caveat, however, is that some writers who had ever had a hallucinatory experience in their lifetime stated that these experiences were noticeably different from their experiences of their characters’ voices.

### 4.1 Limitations

There are a number of limitations to consider when interpreting the results of the present study. This research is the largest mixed methods empirical investigation of a specific aspect of inner experience for which no established vocabulary exists; moreover, it investigates a population who are, by definition, expert in the metaphorical and figurative use of language. Analyses of these data were not, therefore, straightforward. While the online administration of a bespoke phenomenological questionnaire alongside the existing standardised measures was likely key to the successful recruitment of a sample size large enough for statistical analysis, the reliance on unverified self-report, cross-sectional design and inability to follow up with participants are obvious limitations of this research design. Anonymous participation and a corresponding lack of information about the participants’ professional profiles (notably genre and number of publications) make it impossible to speculate as to the existence or significance of differences between writers based either on their expertise or as reflected in their ‘outputs’. We also did not gather data on how long participants had been writing for, which might conceivably affect how their characters were experienced in terms of skill acquisition and automaticity.

Although limiting the study to writers presenting at the Edinburgh International Book Festivals ensured that participants were held in high esteem by members of the literary community, the sample cannot be said to be representative of writers generally. Both the invitation to respondents and the questionnaire were designed in relation to specific research objectives: investigating what writers meant when they reported ‘hearing’ the voices of their characters, and determining whether there were any differences between writers who did and did not report this phenomenon. It is therefore likely that writers who were disinterested in the idea of ‘experiencing’ characters declined to participate, and so it is not possible to estimate the extent to which our results are indicative of the prevalence of such experiences amongst writers more broadly. The study was also prompted by the language that some writers use in relation to their characters (e.g. ‘speaking’, ‘talking’, etc.), and so may not fully capture the complexity and multi-modality of writers’ experiences of their characters. Moreover, those aspects of writers’ experiences which only emerged during thematic analysis (such as experiential overlap and physical acting out) may be somewhat underreported, given that we did not directly ask about these experiences. While this is a possibility, asking direct questions can also lead to important facets of inner experience being missed: it can prompt demand characteristics, and can close down avenues of investigation by encouraging yes or no answers (see Woods et al., 2015, for an example of a similar methodological approach in relation to AVHs). For these reasons, we chose to use a range of open and closed questions to elicit as wide a range of responses as possible.

A final concern is the potential for content overlap in the different constructs being measured by the questionnaires. This is particularly so for the VISQ subscale of ‘other people in inner speech’, since it may be the case that authors endorsing these items were simply re-describing the tendency to experience their characters’ voices. The VISQ is aimed more broadly at inner speech experiences in everyday life, and refers to other people, such as family members, rather than anyone who could be construed as fictional or imaginary (McCarthy-Jones & Fernyhough, 2011). Nevertheless, we must treat the finding that writers who hear their characters’ voices received a higher score on this measure with caution. To clarify the issue, future work would need to make more careful distinctions regarding inner speech inside and outside the writing process.

Notwithstanding these limitations, the present study is, to our knowledge, the only survey of writers’ experiences of their characters which attempts to address the phenomenological complexity of these experiences within a large professional sample. Our results reveal a noticeable degree of heterogeneity which further research into this phenomenon should take into account. First, it would appear that while a small subset of writers do have pronounced experiences of their characters as entirely separate and self-determining agents, they are not particularly representative of writers’ experiences in general, not least because the agency and alterity of characters did not necessarily go hand in hand. Second, while there do seem to be some areas of overlap between writers’ experiences of their characters and analogous experiences of non-actual entities, there are also important phenomenological differences which should perhaps make us wary of relying on a singular explanatory model. Above all, the complexity of writers’ experiences of their characters highlights the need for a more refined conceptual framework for understanding our interactions with actual and non-actual agents in general, with implications for the wider field of social cognition. Many characters may indeed talk back, but not all will; systematic investigation of this phenomenon in a range of writers will allow for a fuller exploration of the boundaries of social cognition, imagination, and the sense of agency.

## CRediT authorship contribution statement

**John Foxwell:** Methodology, Writing - original draft, Writing - review & editing. **Ben Alderson-Day:** Conceptualization, Methodology, Software, Writing - original draft, Writing - review & editing, Funding acquisition. **Charles Fernyhough:** Conceptualization, Methodology, Writing - review & editing, Funding acquisition. **Angela Woods:** Conceptualization, Methodology, Writing - original draft, Writing - review & editing, Supervision, Funding acquisition.

## Footnotes

1 For example, one poet stated: ‘I can hear them talking in my head, and it’s a strange hybrid of me and a separate personality’ (*R 62*), while a historical biographer wrote that ‘It is a real challenge to imagine someone speaking to you from across the centuries […] It’s an extraordinary experience when it happens and can be almost like a private interview with, say, one of the world’s greatest conquerors’ (*R 181*). As with fiction writers, there was substantial variation within these groups, but neither was large enough to allow for subdividing them further.

2 The 2014 survey used a 5-point Likert scale, the results of which were scaled up to match with the 2018 survey. The additional response options were added in the 2018 survey as part of the development of the VISQ-R (Alderson-Day et al., 2018).

3 As Wilkinson & Bell put it, according to this view ‘it would be possible to experience something as an agent *prior* to being consciously aware of any of its perceivable properties’ (Wilkinson & Bell, 2016, p.113).
